# Polymorphisms of the *STAT4* gene in the pathogenesis of tuberculosis

**DOI:** 10.1042/BSR20180498

**Published:** 2018-08-29

**Authors:** Shouquan Wu, Minggui Wang, Yu Wang, Miaomiao Zhang, Jian-Qing He

**Affiliations:** Department of Respiratory and Critical Care Medicine, West China Hospital, Sichuan University, Chengdu, Sichuan, China

**Keywords:** genetic, inflammatory, STAT4, susceptibility, tuberculosis

## Abstract

The signal transducer and activator of transcription 4 (*STAT4*) gene encodes a transcription factor that transmits signals induced by several cytokines which play critical roles in the development of autoimmune and chronic inflammatory diseases. In the present study, we have investigated the association between *STAT4* polymorphisms and a predisposition to *Mycobacterium tuberculosis* (MTB) infection and pulmonary tuberculosis (PTB). In the present study, a total of 209 cases of PTB, 201 subjects with latent TB infection (LTBI), and 204 healthy controls (HC) were included. Logistic regression analyses were used to calculate *P*-values, odds ratios (ORs), and 95% confidence intervals (CIs) for assessing the association between single nucleotide polymorphisms (SNPs) and disease risk. We used Bonferroni correction to adjust the *P*-values. Genotyping was conducted using the improved multiplex ligase detection reaction (iMLDR) method. For the rs7574865 polymorphism, the GT genotype is less frequent in the LTBI group compared with HC (*P*=0.028, OR = 0.62; 95%CI: 0.40–0.95). In addition, the prevalence of the rs897200 CC genotype was lower in the PTB cases compared with LTBI individuals (*P*=0.039, OR = 0.54; 95%CI: 0.30–0.97). However, no SNPs within *STAT4* were associated with PTB or LTBI after Bonferroni correction. Our study demonstrated that *STAT4* variants were not related to LTBI and PTB.

## Introduction

Tuberculosis (TB) continues to be a leading cause of morbidity and mortality in low-income and middle-income countries. In 2016, it was reported that almost 895000 people were diagnosed with TB, and 51800 people died from this disease in China [1]. Recent epidemiologic data demonstrated that approximately 1.7 billion people are infected with *Mycobacterium tuberculosis* (MTB) [2]. However, only 5–15% of infected individuals will develop TB during their lifetime [3]. It remains unknown why only a minority of infected subjects progress to active disease.

Studies have demonstrated that the specific strain of MTB, environmental factors, and host genetics could explain why the incidence of TB is different amongst particular races, geographic areas, genders, and age groups [4,5]. Genetic factors have been related to the progression of TB. Evidence from twin studies indicated that host genetics is involved in the progression of TB [6] and estimates of the heritable component of TB vary from 39 to 71% [7,8].

Innate immune cells and adaptive immune response factors are responsible for the etiopathogenesis of TB. The immune system was associated with protective mechanisms against MTB [9]. Several functional single nucleotide polymorphisms (SNPs) have been shown to modulate susceptibility to infection [10].

Signal transducer and activator of transcription 4 (STAT4) is one of the STAT family members. The *STAT4* gene, located on human chromosome 2q32.2-q32.3, consists of 27 exons and encodes a transcription factor that is expressed in dendritic cells, macrophages, and lymphocytes. STAT4 can transduce signals induced by cytokines such as type I interferons (IFNs), interleukin-12 (IL-12), and IL-23 in monocytes and T cells. STAT4 plays a critical role in the differentiation of T helper (Th) cells to the Th1 phenotype in response to IL-12. It is well-established that the IL-12/IFN-γ axis plays a key role in the elimination and control of MTB [11]. SNPs in the *IL-12RB1* and *IL-12B* genes have been associated with susceptibility to TB [12,13]. STAT4 also promotes the differentiation of Th17 cells, a CD4^+^ T-cell lineage that plays an essential role in autoimmunity-associated inflammation. Th1 and Th17 cells are related to several inflammatory and autoimmune diseases [14].

To date, three studies have been conducted to investigate the relationship between *STAT4* polymorphisms and TB, and one of them demonstrated that *STAT4* promoter region polymorphisms were associated with pulmonary TB (PTB) and may impact *STAT4* expression [15]. However, the other two studies did not find association of TB with *STAT4* variants [16,17].

The aim of the current study was to investigate the role of *STAT4* SNPs in PTB and latent TB infection (LTBI) in Chinese Han patients. To the best of our knowledge, this is the first study to investigate the correlation between *STAT4* polymorphisms and LTBI/TB risk in the Chinese Han population.

## Methods

### Study population

A total of 209 PTB and 415 close contacts of individuals with sputum-positive PTB were enrolled between 2013 and 2014. All the participants were recruited from the West China Hospital of Sichuan University (Sichuan, China), and they were all genetically unrelated to Chinese Han people. PTB cases were all bacteriologically confirmed patients. Ten close contacts who developed PTB during a 1-year follow-up were excluded. We stratified the remaining 405 close contacts of PTB cases into LTBI subjects and healthy controls (HC) depending on IFN γ release assay (IGRA) results, symptoms, chest X-ray, and sputum examination. The definition of close contacts was as follows: (i) shared airspace with a PTB patient for at least 15 h per week for at least 1 week during an infectious period, (ii) shared airspace with a PTB patient for at least 180 h during an infectious period. LTBI and HC individuals had no TB-related symptoms and negative sputum acid-fast bacilli smear for *MTB*. None of the participants was reported to have concomitant chronic obstructive pulmonary disease, HIV infection, hepatitis B virus (HBV), and/or HCV infection, or immune-mediated disorders.

 Venous blood (2–5 ml) was drawn from each participant after they agreed to participate in this research and gave informed consent. The blood sample was collected using EDTA tubes, and then stored in a −80°C freezer until further investigation. We extracted the DNA from the blood using a genomic DNA Purification kit (Axygen Scientific Inc., Union City, CA, U.S.A.) in accordance with the manufacturer’s instructions. The DNA specimens were stored at −80°C for further genotyping. The present study was approved by the ethical committee of the West China Hospital Institutional Review Board.

### SNP selection and genotyping

SNPs were selected based on previous studies of *STAT4* genetic associations with TB [15,16] combined with linkage disequilibrium (LD) information amongst *STAT4* SNPs in the Chinese population obtained from the HapMap database (http://hapmap.ncbi.nlm.nih.gov/index.html.en, HapMap Data Rel 27 Phase II + III, on NCBI B36 assembly, dbSNP b126), using minor allele frequency (MAF) ≥5% and *R^2^* threshold of 0.80, in a region 3000 bp upstream and 2000 bp downstream of *STAT4*. The *STAT4* SNP genotyping was conducted using the improved multiplex ligase detection reaction (iMLDR), with technical support from the Shanghai Genesky Biotechnology Company. Samples (5%) were genotyped in duplicate to check for concordance.

### Statistical analyses

We used χ^2^ tests to examine whether the control groups conformed to the Hardy–Weinberg equilibrium (HWE). An unpaired *t* test was used to check the difference of mean age between controls and cases. Logistic regression analyses under allelic, recessive, and dominant genetic models were employed to calculate 95% confidence intervals (CIs), odds ratios (ORs), and *P*-values to evaluate the relationship between SNPs and TB susceptibility as well as to adjust for age and sex. Haplotype analyses within our dataset were performed using the SHEsis online software platform (http://analysis.bio-x.cn). Power analysis was conducted by using the Power and Sample Size Calculation Software (http://biostat.mc.vanderbilt.edu/PowerSampleSize). *P*-values were from two-tailed tests and statistical significance was set at *P*<0.05. Bonferroni correction was used to adjust the *P*-values for multiple comparisons. Thus, a *P*-value <0.0125 (0.05/4) was considered statistically significant for multiple comparisons. All analyses were conducted by using the Statistical Package for the Social Sciences (SPSS, SPSS Inc., Chicago, IL, U.S.A.).

## Results

### Characteristics of study subjects

The characteristics of the three study groups are shown in Table 1. We recruited 209 PTB cases (107 males and 102 females), 201 LTBI individuals (95 males and 106 females), and 204 HC (93 males and 111 females) in the present study. The mean of age was 38.76 ± 16.97 years for the PTB group, 49.09 ± 15.91 years for the LTBI group, and 45.71 ± 14.90 for the HC. There was no significant difference in the sex distribution between the groups. However, the distribution of age was significantly different between the three groups.

**Table 1:** Demographic and clinical characteristics of the study groups

	PTB (*n*=209)	LTBI (*n*=201)	HC (*n*=204)	PTB compared with LTBI *P-*value	LTBI compared with HC *P*-value
Age, mean ± S.D.	38.76 ± 16.97	49.09 ± 15.91	45.71 ± 14.90	<0.001	0.027
Male, *n* (%)	107 (0.51)	83 (0.48)	84 (0.46)	0.539	0.750
Symptoms and signs					
Cough, *n*	153				
Hemoptysis, *n*	31				
Dyspnea, *n*	51				
Night sweats, *n*	68				
Thoracalgia, *n*	31				
Fever, *n*	82				
Lung rales, *n*	37				

### Characteristics of SNPs

Four *STAT4* SNPs (rs7574865, rs4853542, rs1031509, and rs897200) were selected for the present study. Two of these polymorphisms were selected based on the study by Sabri et al. [15], which demonstrated that rs1031509, rs7572482, and rs897200 were associated with PTB in a Moroccan population. rs7572482 and rs897200 were in high LD in the Moroccan population with an *R^2^* of 0.96, and they were also in high LD in Chinese Han people with *R^2^* of 1.00, so we only selected rs1031509 and rs897200 for our study. We also selected a polymorphism (rs7574865) that was shown to be associated with level of *STAT4* mRNA and protein expression [18]. Finally, we selected one Tag-SNP (rs4853542) as it was a surrogate for 15 other common SNPs that formed the largest ‘bin’ of *STAT4* SNPs in Chinese Han individuals from the HapMap database. The characteristics of the selected SNPs are shown in Table 2.

**Table 2:** Characteristics of the SNPs

SNPs	Chromosome	Location (bp)	Location in gene	MA	MAF	MA	MAF	HWE *P*-value	
				LTBI		HC		LTBI	HC
rs7574865	2	191, 964, 633	Intron 3	T	0.33	T	0.35	0.291	0.401
rs4853542	2	191, 976, 602	Intron 3	A	0.28	A	0.30	0.936	0.454
rs1031509	2	192, 010, 189	Intron 3	T	0.36	T	0.32	0.840	0.979
rs897200	2	192, 017, 771	5′-flanking	C	0.41	C	0.44	0.922	0.871

Abbreviation: MA, minor allele.

### Association between *STAT4* polymorphisms and LTBI or TB susceptibility

The genotype distribution of the four SNPs is shown in Table 3. The frequency of the *STAT4* rs7574865 GT genotype was lower in the LTBI compared with HC (*P*=0.028, OR = 0.62; 95%CI: 0.40–0.95). In addition, the rs897200 CC genotype in a recessive model was less prevalent in the PTB compared with LTBI group (*P*=0.039, OR = 0.54; 95%CI: 0.30–0.97). However, none of the SNPs in *STAT4* was significantly associated with PTB or LTBI after Bonferroni correction.

**Table 3:** Association between *STAT4* genotypic/allelic frequencies and LTBI/PTB

SNPs		PTB (%), *n*=209	LTBI (%), *n*=201	HC (%), *n*=204	PTB compared with LTBI		LTBI compared with HCS	
					P*	OR (95%CI)*	P^*^	OR (95%CI)^*^
rs7574865G > T	Genotype							
	GG	96 (45.9)	96 (48.0)	83 (40.7)				
	GT	86 (41.1)	78 (39.0)	101 (49.5)	0.464	1.18 (0.76–1.85)	0.028	0.62 (0.40–0.95)
	TT	27 (12.9)	26 (13.0)	20 (9.8)	0.981	0.99 (0.53–1.87)	0.637	1.17 (0.61–2.27)
	Alleles							
	G	278 (66.5)	270 (67.55)	267 (65.4)				
	T	140 (33.5)	130 (32.5)	141 (34.6)	0.737	1.03 (0.76–1.41)	0.506	0.91 (0.67–1.22)
Genetic model	Dominant				0.610	1.11 (0.74–1.68)	0.097	0.71 (0.48–1.06)
	Recessive				0.723	0.90 (0.48–1.65)	0.229	1.47 (0.78–2.76)
rs4853542G > A	Genotype							
	GG	99 (47.4)	104 (52.0)	104 (51.0)				
	AG	90 (43.1)	79 (39.5)	78 (38.2)	0.543	1.14 (0.74–1.76)	0.947	0.99 (0.65–1.50)
	AA	20 (9.6)	17 (8.5)	22 (10.8)	0.463	1.32 (0.63–2.74)	0.441	0.76 (0.38–1.53)
	Alleles							
	G	288 (68.9)	287 (71.8)	286 (70.1)				
	A	130 (31.1)	113 (28.3)	122 (29.9)	0.401	1.15 (0.83–1.57)	0.551	0.91 (0.67–1.24)
Genetic model	Dominant				0.453	1.17 (0.78–1.76)	0.757	0.94 (0.63–1.39)
	Recessive				0.560	1.24 (0.61–2.53)	0.441	0.77 (0.39–1.50)
rs1031509G > T	Genotypes							
	GG	85 (40.7)	80 (40.0)	94 (46.1)				
	GT	102 (48.8)	96 (48.0)	88 (43.1)	0.489	1.17 (0.75–1.80)	0.295	1.25 (0.82–1.90)
	TT	22 (10.5)	24 (12.0)	22 (10.8)	0.786	0.91 (0.45–1.82)	0.442	1.29 (0.67–2.49)
	Alleles							
	G	272 (65.1)	256 (64.0)	276 (67.6)				
	T	146 (34.9)	144 (36.0)	132 (32.4)	0.903	1.02 (0.75–1.38)	0.315	1.16 (0.87–1.56)
Genetic model	Dominant				0.587	1.12 (0.74–1.71)	0.274	1.25 (0.84–1.86)
	Recessive				0.568	0.83 (0.44–1.57)	0.674	1.14 (0.62–2.12)
rs897200T > C	Genotypes							
	TT	72 (34.4)	71 (35.5)	65 (31.9)				
	CT	113 (54.1)	94 (47.0)	97 (47.5)	0.576	1.14 (0.72–1.79)	0.500	0.86 (0.55–1.34)
	CC	24 (11.5)	35 (17.5)	42 (20.6)	0.118	0.59 (0.31–1.14)	0.497	0.82 (0.46–1.45)
	Alleles							
	T	257 (61.5)	236 (59.0)	227 (55.6)				
	C	161 (38.5)	164 (41.0)	181 (44.4)	0.261	0.84 (0.63–1.13)	0.414	0.89 (0.67–1.18)
Genetic model	Dominant				0.927	0.98 (0.64–1.51)	0.436	0.85 (0.56–1.29)
	Recessive				0.039	0.54 (0.30–0.97)	0.601	0.87 (0.53–1.45)

* Adjusted by age and sex.

Haplotype analyses suggested that no significant associations of the haplotypes with PTB/LTBI were found after Bonferroni correction (Table 4).

**Table 4:** Haplotypes of the *STAT4* genes and their distributions in the three groups

Haplotype	LTB compared with PTB				HC compared with LTBI			
	Case (%), *n*=418	Control (%), *n*=400	*P*	OR (95%CI)	Case (%), *n*=400	Control (%), *n*=408	*P*	OR (95%CI)
GAGC	18.40 (4.4)	25.25 (6.3)	0.266	0.71 (0.38–1.31)	25.25 (06.3)	41.48 (10.2)	0.038	0.58 (0.35–0.97)
GAGT	74.07 (17.7)	69.62 (17.4)	0.749	1.06 (0.74–1.52)	69.62 (17.4)	51.40 (12.6)	0.073	1.43 (0.97–2.11)
GATT	23.61 (5.6)	16.78 (4.2)	0.290	1.41 (0.74–2.68)	16.78 (4.2)	19.53 (4.8)	0.639	0.85 (0.44–1.66)
GGGC	75.66 (18.1)	81.03 (20.3)	0.567	0.90 (0.64–1.28)	81.03 (20.3)	79.16 (19.4)	0.872	1.03 (0.73–1.46)
GGGT	19.15 (4.6)	11.30 (2.8)	0.158	1.71 (0.81–3.61)	11.30 (2.8)	23.49 (5.8)	0.034	0.47 (0.23–0.96)
GGTT	67.11 (16.1)	66.02 (16.5)	0.986	1.00 (0.69–1.46)	66.02 (16.5)	51.93 (12.7)	0.161	1.32 (0.90–1.96)
TGGC	63.16 (15.1)	57.72 (14.4)	0.648	1.09 (0.74–1.61)	57.72 (14.4)	57.28 (14.0)	0.968	1.01 (0.68–1.50)
TGGT	14.72 (3.5)	11.05 (2.8)	0.485	1.33 (0.60–2.93)	11.05 (2.8)	16.17 (4.0)	0.318	0.67 (0.31–1.47)
TGTT	48.20 (11.5)	59.88 (15.0)	0.199	0.77 (0.51–1.15)	59.88 (15.0)	57.96 (14.2)	0.851	1.04 (0.70–1.54)
Other pooled*	13.92 (3.3)	1.36 (0.3)			1.33 (0.3)	9.58 (2.4)		

* Subjects with haplotype frequencies <0.03 both in cases and controls were pooled in this category.

### Power analysis

We conducted a power analysis to assess the sample size in our study. We used reported ORs of 1.49, 1.69, and 1.89 (minimum, median, and maximum) [15] to calculate the power of the sample size for each SNP. The results indicated that the sample size provides sufficient power (>80%) to draw the conclusion with OR = 1.69 or above (Table 5).

**Table 5:** Power of the study with different ORs in an allelic model

SNPs	MAF		Power in PTB compared with LTBI			Power in LTBI compared with HC		
	LTBI	HC	OR = 1.49	OR = 1.69	OR = 1.89	OR = 1.49	OR = 1.69	OR = 1.89
rs7574865	0.325	0.346	0.785	0.954	0.994	0.790	0.956	0.994
rs4853542	0.283	0.299	0.760	0.944	0.991	0.766	0.946	0.992
rs1031509	0.360	0.324	0.799	0.959	0.995	0.780	0.952	0.993
rs897200	0.410	0.444	0.810	0.963	0.995	0.808	0.961	0.995

## Discussion

Most previous studies of genetic factors in TB focussed on the association between gene polymorphisms and TB using control subjects that included LTBI and uninfected individuals. However, few studies have identified candidate genes related to LTBI and/or TB. In the present study, we designed three study groups including HC without TB infection, LTBI, and PTB to identify the risk factors for both LTBI and TB, and hence, to find genetic markers specific for TB developmental stages. We demonstrated that polymorphisms in *STAT4* were not associated with PTB or LTBI.

Evidence from infections in immunocompromised patients, twin comparisons, candidate gene, and genome-wide association studies indicates that host genetic factors affect TB susceptibility [19–23]. The recent literature has focussed on the role of the host innate immune system in influencing the susceptibility to TB [21]. Although some critical factors for MTB resistance have already been identified, it is necessary to further investigate the fine-tuning of the immune response to provide better targets for therapeutic manipulation of the immune system. *STAT4* polymorphisms have been associated with various diseases, e.g. *STAT4* rs8179673 was demonstrated to be a protective factor against HBV infection [24]. rs7582694 has been associated with immune-related diseases such as multiple sclerosis [25], systemic lupus erythematosus [25], and type-1 autoimmune hepatitis [26]. Furthermore, studies of different populations indicated that rs11889341/rs10181656 in *STAT4* were associated with dilated cardiomyopathy [27] and neuromyelitis optica spectrum disorders [28].

To our knowledge, only three TB association studies have investigated *STAT4* as a candidate gene. Sabri et al. [15] suggested that three *STAT4* promoter region polymorphisms (rs1031509, rs7572482, and rs897200) were associated with PTB in a Moroccan population. Hijikata et al. [17] conducted a PTB association study, focussing on a single *STAT4* microsatellite marker, without any significant results. Sanchez et al*.* [16] assessed the association of genetic polymorphisms in transcription factor genes, including *STAT4*, with susceptibility/resistance to PTB and the results also indicated that no *STAT4* SNP was related to TB. A previous Chinese study found that the subjects with the rs897200TT genotype had significantly higher *STAT4* mRNA levels in peripheral blood mononuclear cells and skin cells than CC individuals [29]. Reporter gene assays also demonstrated that promoter activity was significantly increased in cells carrying the rs897200 A allele compared with cells carrying the C allele [29]. In the present study, we demonstrated that the rs897200 and rs1031509 polymorphisms were not associated with PTB and LTBI after Bonferroni correction.

In the study of Sabri et al. [15], a strong association between rs897200 and TB was identified, with the C allele being a risk factor for TB. In contrast, rs897200 C was more common in the control group in our study. Furthermore, we found no significant association between any SNPs at this locus and PTB. There are several possible explanations for these discrepancies. First, Sabri et al*.* [15] did not differentiate between LTBI subjects and those without TB infection. Previous studies have identified genes associated with LTBI but not active TB [30], as well as genes related to active TB but not LTBI [31]. As others have suggested, differences in study design could influence the results of genetic association studies [32]. Second, differences in MAF between the two ethnic groups could be another cause of the conflicting results. Third, different strains of MTB interacting with the host immune response may result in distinct clinical phenotypes [33] and could also explain the inconsistent results.

Although rs7574865 was not associated with TB in a Colombian population [17], it was reported to be a functional SNP with the rs7574865T allele associated with higher *STAT4* mRNA and protein expression [18]. rs7574865 has been associated with many disorders such as inflammatory bowel disease and HBV-related hepatocellular carcinoma [34,35]. Therefore, rs7574865 was selected in our study. Consistent with a previous study, we found that rs7574865 was not a causal polymorphism for PTB/LTBI. To date, there have been no studies that have examined the association between rs4853542 and any phenotype. Our results suggest that this SNP may not be associated with LTBI/TB. In addition, power analysis revealed that the sample size of the present study was sufficient to draw meaningful conclusion for each SNP. Further studies are warranted to verify our results.

Despite our study demonstrating that *STAT4* polymorphisms were not associated with PTB/LTBI, confounding factors in this genetic association study should be taken into consideration. Generally, population structure is an important source of confounding in genetic association studies [36,37]. It was estimated that population structure partly contributed to a significant 11.2% inflation of test statistics [37]. In order to reduce the possibility of spurious results caused by such confounding, we selected case and control subjects from the southwest of China and all the subjects were Han Chinese. Indeed, matching each case with a control from same subpopulation could avoid the problem of spurious association results [37]. Like population structure, cryptic relatedness could also have a confounding effect on association results. This confounding usually arises in studies conducted in smaller groups of individuals. Thus, choosing samples of the same ethnic group from a large population (i.e. the southwest of China) could potentially solve the problem of spurious results caused by both population structure and cryptic relatedness. In addition, the genotype distributions of all four *STAT4* SNPs conformed to HWE, which may minimize the likelihood of cryptic relatedness in our study population [38].

## Conclusion

In summary, we found that *STAT4* polymorphisms were not associated with LTBI or PTB, which represents a gene-based investigation of this candidate in a population not previously studied. Our results may help further research on the potential role of the STAT4 pathway in human immune responses to MTB infection and progression to active TB.

## Abbreviations

CI: confidence interval
HBV: hepatitis B virus
HC: healthy control
HWE: Hardy–Weinberg equilibrium
IFN: interferon
IL: interleukin
LD: linkage disequilibrium
LTBI: latent tuberculosis infection
MAF: minor allele frequency
MTB: *Mycobacterium tuberculosis*
OR: odds ratio
PTB: pulmonary tuberculosis
SNP: single nucleotide polymorphism
STAT4: signal transducer and activator of transcription 4
TB: tuberculosis
Th: T helper
